# The added value of chlamydia screening between 2008-2010 in reaching young people in addition to chlamydia testing in regular care; an observational study

**DOI:** 10.1186/s12879-014-0612-2

**Published:** 2014-11-18

**Authors:** Geneviève AFS van Liere, Nicole HTM Dukers-Muijrers, Jan EAM van Bergen, Hannelore M Götz, Frans Stals, Christian JPA Hoebe

**Affiliations:** Department of Sexual Health, Infectious Diseases and Environmental Health, South Limburg Public Health Service, Geleen, 6160 HA The Netherlands; Department of Medical Microbiology, School of Public Health and Primary Care (CAPHRI), Maastricht University Medical Centre (MUMC+), Maastricht, 6202 AZ The Netherlands; STI AIDS Netherlands, Keizersgracht 390, Amsterdam, 1016 GB The Netherlands; Department of Infectious Diseases Control, Municipal Public Health Service Rotterdam-Rijnmond, Rotterdam, 3000 LP The Netherlands; Department of Medical Microbiology, Atrium Medical Centre Parkstad, Heerlen, 6401 CX The Netherlands

**Keywords:** Chlamydia screening, General practitioners, STI clinic, Medical specialists, Chlamydia, Testing, Newly reached participants, Regular care, Young individuals, Hidden

## Abstract

**Background:**

Internet-based Chlamydia Screening Implementation (chlamydia screening programme) was introduced in the Netherlands in 2008-2010 to detect and treat asymptomatic infections and to limit ongoing transmission through annual testing and treatment of *Chlamydia trachomatis* in young people (16-29 years). This population-based screening may be less effective when addressing individuals who are already covered by regular care, instead of addressing a hidden key population without chlamydia testing experience in regular care. This study had two aims: (1) to assess the rate and determinants of newly reached (i.e. not previously tested in 2006-2010) participants in the chlamydia screening programme, and (2) to assess the chlamydia positivity in these newly reached participants.

**Methods:**

This observational matching study included all chlamydia tests performed in subjects aged 16-29 years in eastern South Limburg in the Netherlands (population 16-29 years:41,000) between 2006-2010. Testing was conducted during the systematic chlamydia screening programme (2008-2010), at a sexually transmitted infections clinic (STI clinic), by general practitioners (GPs), and by medical specialists as reported by the medical laboratory serving the region. Data were matched between testing services on individual level. The study population included all participants who were tested at least once for chlamydia by the chlamydia screening programme. Participants were included at their first chlamydia screening participation.

**Results:**

In the chlamydia screening programme, 80.7% (4298/5323) of participants were newly reached, others were previously tested by the STI clinic (5.7%, n=304), GPs (6.2%, n=328), medical specialists (3.5%, n=187) or a combination of providers (3.9%, n=206). Chlamydia prevalence was similar in newly reached participants (4.8%, 204/4298) and participants previously tested (4.5%, 46/1025, P=0.82). Independent determinants for being a newly reached participant were male gender (men OR 2.9; 95% CI 2.5-3.4) and young age <21 years (versus 25-29 years OR 1.8; 95% CI 1.5-2.2).

**Conclusions:**

The majority of the chlamydia screening programme participants have not been tested by regular care, and show similar chlamydia prevalence as those previously tested. Thereby population-based chlamydia screening adds to the existing regular care by testing young individuals hidden to current regular care.

**Electronic supplementary material:**

The online version of this article (doi:10.1186/s12879-014-0612-2) contains supplementary material, which is available to authorized users.

## Background

Chlamydia is the most prevalent treatable sexually transmitted infection worldwide and has major public health consequences, especially in young women [1]. Early detection and treatment is warranted to limit the spread of infection and to reduce sequelae in infected individuals. A possible complication is pelvic inflammatory disease, where *Chlamydia trachomatis* ascends to the upper genital tract causing tubal factor infertility and ectopic pregnancy. In the Netherlands the regular care for testing and treatment of chlamydia is provided by general practitioners (GPs), sexually transmitted infections (STI) clinics and after referral by medical specialists (mainly gynaecologists) [2]. Thus regular care providers are GP’s, STI clinics and medical specialists. Systematic population-based internet chlamydia screening was initiated in 2008 and aimed to improve case finding to prevent sequelae and to reduce population prevalence by annual testing and treatment of people aged 16-29 years in three regions in the Netherlands. The choice for targeting 16 to 29-year-olds in the additional chlamydia screening programme was based on the highest burden of chlamydia infection among these young people [3],[4]. After a postal invitation, home sampling kits for urogenital testing (urine or vaginal swab) could be requested through a website. Treatment and partner notification were done by the GP or at a STI clinic [5]. The rationale for the chosen approach in the chlamydia screening programme were based on existing evidence for screening programmes, costs, flexible communication, easy adaptation of the screening in time and the possibility of easy expansion to other geographic areas in the future [6]. Moreover, acceptability of the screening method using internet was high [7].

Keystones for an effective large-scale screening programme are achieving adequate levels of participation [8] and capturing substantial numbers of new (chlamydia positive) participants in addition to regular care like STI clinics and general practitioners (GPs). To understand and interpret the outcome of chlamydia screening, knowing who takes part in the chlamydia screening programme is essential [9]. By assessing the totality of chlamydia testing practices, it becomes clear whether the chlamydia screening programme reached persons already served by regular care or a hidden key population without chlamydia testing experience in regular care. Chlamydia screening would become less effective when reaching those who were already tested by regular care. Therefore chlamydia screening should target this hidden key population to prevent chlamydia sequelae in individuals and to diminish further spread of chlamydia in the population additional to the efforts in regular care. Publications assessing the totality of chlamydia testing practices including additional chlamydia screening in a programme are limited [10],[11], as such an assessment is frequently hampered by unavailability of data or unmatchable data sources. Therefore the rationale of this study was to bridge this gap and evaluate this second keystone by using a near complete large data collection of matched test data sources in the target region for the chlamydia screening programme. This study had two aims: (1) to assess the rate and determinants of newly reached men and women aged 16-29 years in the chlamydia screening programme in eastern South Limburg, and (2) to assess the chlamydia positivity in these newly reached men and women.

## Methods

### Systematic chlamydia screening in the Netherlands

The Dutch chlamydia screening programme used systematic-population-based internet chlamydia screening in three regions of the Netherlands, including the eastern South Limburg study area. The intervention was implemented by means of a stepped wedge design, with sequential roll out to geographical clusters of potential participants in a randomly determined order over time so that, by the end of the three year study period, each cluster had been invited at least once. The stepped wedge design was chosen to be able to evaluate participation and effectiveness over several rounds of screening. In three screening rounds from 2008-2010, all men and women aged 16-29 years who were listed in the study area’s municipal population register (n = 41,000, 2010) were sent an invitation letter [5]. In our study area South Limburg, eligibility for chlamydia testing within the chlamydia screening programme depended on an individual’s chlamydia risk score. This risk score was based on answers to an eight-item risk questionnaire (i.e. age, place of residence, education level, condom use at last intercourse, number of lifetime sex contacts, ethnic background, having a new sexual partner in the last 6 months and symptoms) [12]. When a person was eligible, home sampling kits for urogenital testing could be requested through a website (www.chlamydiatest.nl). Chlamydia screening participants could provide additional data via an optional electronic general questionnaire (hereafter questionnaire).

### Study design; data collection and matching

**Figure 1:**
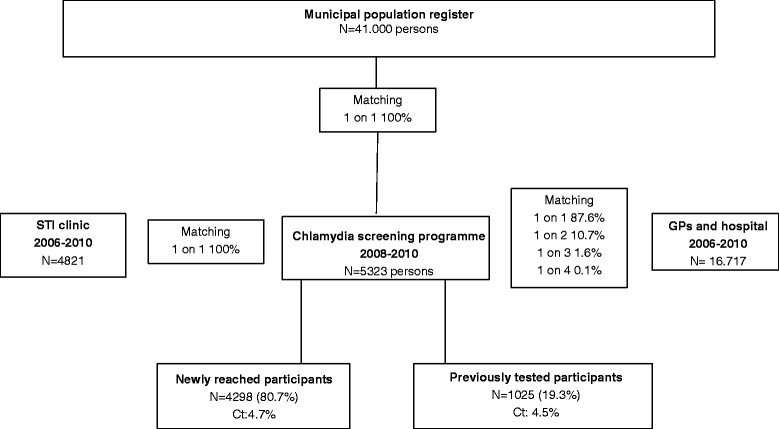
**Flow diagram of the data matching and study procedures.** Data from the STI clinic, the chlamydia screening programme, GPs and the hospital were matched at individual level to the municipal population register. Data from the STI clinic and the chlamydia screening programme were matched uniquely. Data from GPs and hospital were matched by probabilistic algorithmic matching. The study population included all participants who were screened at least once for chlamydia (n = 5395).

Three data sources were used for all the men and women aged 16-29 years who were tested for chlamydia in the study area between 2006 and 2010: the chlamydia screening programme, an STI clinic, and the medical laboratory [13]. Data from GPs and medical specialists (mainly gynaecologists) were obtained from the regional medical microbiology laboratory covering the study area (>95%). Data from the STI clinic were retrieved from our public health STI clinic’s medical records comprising confirmed test results. The basis for data matching was the municipal population register, which included men and women aged 16-29 years who were invited for and tested in the chlamydia screening programme. Data from GPs and medical specialists were uniquely matched to the register on personal level by part of the last name, month of birth, year of birth, sex, and postal code. Part of the test records had identical part of the last name, month of birth, year of birth, sex, and postal code. These records were considered to belong to the same individual, and were matched to the identical municipal population register record. For chlamydia screening participants, a ranking order was assigned to all matched records from GPs and medical specialists (medical laboratory) for sensitivity analyses. Data from the STI clinic were all matched uniquely to the register based on the whole name and date of birth (Figure 1).

### Study population

The study population included all participants who were tested at least once for chlamydia by the chlamydia screening programme. Participants were included at their first chlamydia screening participation. Data on sex, age, and test result were available for all participants, data on same-sex behaviour, symptoms and number of sex partners in the past six months was only available for participants who filled in questionnaire. Variables used for analyses were sex, age (≤21, 22-24, 25-29 years (reference), based on tertiles), test result, nationality (Western vs. non-Western), same-sex behaviour (men who have sex with men, heterosexual men and women (reference)), symptoms, and number of sex partners in the past six months (1, 2, ≥3, based on tertiles).

### Chlamydia trachomatis diagnosis

Specimens tested by the chlamydia screening programme and at the STI clinic came from mostly self-collected vaginal swabs and urine. GPs and medical specialists used mostly clinician-collected urethral and cervical swabs. The STI clinic, GPs, and medical specialists used SDA and PCR for *Chlamydia trachomatis* testing (Becton Dickinson ProbeTec ET system, Maryland, USA and from 6-1-2010 Abbott M2000, Illinois, USA). The chlamydia screening programme used PCR (Roche Cobas Taqman, California, USA). All tests were performed according to the manufacturers’ protocols.

### Analysis

At the first chlamydia screening participation, we assessed whether participants were previously tested by one or more regular care providers between 2006-2010 based on matched data. Participants who were not previously tested were defined as ‘newly reached participant’. The proportion of positive chlamydia tests was compared between newly reached participants and previously tested participants using a Chi square test; adjusting for age and gender did not change the results. Logistic regression analysis was performed: being a newly reached participant was used as the outcome to assess the association with the determinants age, sex, nationality and test result. To assess the association between newly reached participant and determinants from the questionnaire, the second analysis was restricted to participants with a questionnaire. Assessed determinants included age, sex, nationality, sexual preference, symptoms, number of sex partners in the past six months and test result. To test for selection bias, age, sex, nationality, test result and newly reached participant were compared between participants with and without questionnaire using Chi square test. Independent determinants were assessed by multivariable analyses using stepwise backward selection. Interactions terms were added in the multivariable model but none were statistically significant and they were not included in the final model. All analyses were adjusted for year of invitation for the chlamydia screening. A P value <0.05 was considered to be statistically significant. Analyses were performed using the SPSS package version 20 (IBM Inc. Somers, New York, USA).

### Ethics statement

Participants, including minors (16-18 years-old), provided written consent to participate in this study, including consent for further research. No written consent was obtained from next of kin, caretakers, or guardians on behalf of the minors enrolled in the study. The Medical Ethics Committee of the VU University Amsterdam (Identification number 2007/239) approved the chlamydia screening trial. The Medical Ethics Committee of Maastricht University (Identification number 12-4-042) approved the study, including the consent procedure and data matching.

## Results

### Data matching

The STI clinic database comprised 4821 young people (16-29 years), the GP and hospital database comprised 16.717 young people. In total 41.000 young people were invited for chlamydia screening, 5395 participated at least once (13.2%). Participants who were 16 years old at the first chlamydia screening (n = 72) were excluded from analysis because there were no previous testing data available from regular care providers. The study population comprised 5323 participants (participation 13.0%). Of all test records matched from GPs and medical specialists with the chlamydia screening participants in the municipal population register (n = 1287), 87.6% (n = 1127) were matched uniquely, 10.7% (n = 138) were matched 1 on 2, 1.6% (n = 21) were matched 1 on 3, and 0.1% (n = 1) was matched 1 on 4. Data from the STI clinic were all uniquely (1 on 1) matched based on first name, last name, date of birth, sex, and postal code (n = 422). In total, 90.6% (1549/1709) of data were uniquely matched (1 on 1) (Figure 1).

### Study population

The largest proportion of participants consisted of participants 25-29 years of age, followed by participants of 22-24 years of age. Two thirds of participants were women, and the majority had Western nationality (>95%). In total, 59.1% (n = 3162) of participants filled in the questionnaire. Participants without questionnaire were older, more often men, more often had non-Western nationality and had lower chlamydia prevalence compared to participants with questionnaire (Table 1).

**Table 1:** **Characteristics of chlamydia screening programme participants without and with questionnaire**

Variables	Participants without questionnaire N = 2161 % (N)	Participants with questionnaire N = 3162 % (N)	Total N = 5323 % (N)
Age			
≤ 21	24.2 (523)	28.7 (906)*	26.8 (1429)
22-24	36.1 (781)	34.2 (1081)	35.0 (1862)
25-29	39.7 (857)	37.2 (1175)	38.2 (2032)
Sex			
Men	42.5 (919)	29.1 (919)*	34.5 (1838)
Women	57.5 (1242)	70.9 (2243)	65.5 (3485)
Nationality			
Non- Western	3.8 (81)	2.0 (64)*	2.7 (145)
Chlamydia			
Yes	2.9 (63)	5.9 (181)*	4.7 (250)
Newly reached participant			
Yes	80.8 (1746)	80.7 (2552)	80.7 (4298)

*P < 0.01.

### Newly reached participants

In the chlamydia screening programme, 80.7% (4298/5323) of participants were newly reached (not previously tested). The other participants had been tested previously at least once: 5.7% (304/5323) by the STI clinic, 6.2% (328/5323) by GPs, 3.5% (187/5323) by medical specialists, and 3.9% (206/5323) by a combination of providers. The proportion of newly reached participants was comparable in participants with and without questionnaire (Table 1). Of all previous tests, 2.7% (n = 28) were tested within 3 months before chlamydia screening participation.

### Determinants newly reached participants

In multivariable analyses, independent determinants for a newly reached participant were male gender and young age. Nationality and test result were not associated. In restricted analyses including questionnaire data, further identified independent determinants for newly reached participants were young age, having had 1 or 2 sex partners (compared to 3 sex partners), being a man who had sex with men (MSM), and being a heterosexual man (compared to women). Sex, symptoms, or test result (see section below) were not associated (Table 2).

**Table 2:** **Characteristics of newly reached participants and previously tested participants**

Variables	Newly reached participants N = 4298 % (N)	Previously tested N = 1025 % (N)	Unadjusted OR(95%CI)	Adjusted OR(95%CI)	P value
Age					
≤ 21	28.7 (1233)	19.1 (196)	1.63 (1.4-2.0)	1.8 (1.5-2.2)	<0.001
22-24	33.8 (1451)	40.1 (411)	0.9 (0.8-1.1)	1.0 (0.8-1.1)	0.56
25-29	37.6 (1614)	40.8 (418)	1	1	
Sex					
Men	38.3 (1646)	18.7 (192)	2.7 (2.3-3.2)	2.9 (2.5-3.4)	<0.001
Women	61.7 (2652)	81.3 (833)	1	1	
Chlamydia					
Yes	4.8 (204)	4.5 (46)	1.1 (0.8-1.5)	ns	
No			1		
Nationality					
Non-Western	2.6 (111)	3.3 (34)	0.8 (0.5-1.2)		
Western	96.7 (990)	97.4 (4159)	1	ns	
**Restricted analyses**					
Age					
≤ 21	30.7 (783)	20.2 (123)	1.7 (1.4-2.2)	1.7 (1.3-2.2)	<0.001
22-24	33.0 (842)	39.2 (239)	0.9 (0.8-1.2)	1.0 (0.8-1.2)	0.78
25-29	36.3 (927)	40.7 (248)	1	1	1
Sex					
Men	32.4 (827)	15.1 (92)	2.7 (2.1-3.4)	ns	
Women	67.6 (1725)	84.9 (518)	1		
Chlamydia					
Yes	5.9 (150)	6.1 (37)	1.0 (0.7-1.4)	ns	
No	94.1 (2402)	93.9 (573)	1		
Sexual preference					
Men who have sex with men	1.7 (39)	0.9 (5)	2.3 (0.9-5.9)	4.0 (1.6-10.6)	<0.01
Heterosexual men	30.4 (715)	13.8 (77)	2.8 (2.2-3.6)	3.3 (2.6-4.3)	<0.001
Women	67.9 (1598)	85.3 (477)	1	1	
Sex partners					
1	73.3 (1713)	63.8 (353)	1.8 (1.4-2.4)	2.6 (1.9-3.4)	<0.001
2	15.9 (371)	19.2 (106)	1.3 (0.9-1.8)	1.7 (1.2-2.4)	<0.01
≥3	10.8 (253)	17.0 (94)	1	1	
Symptoms					
Yes	2.4 (62)	3.0 (18)	1.3 (0.8-2.1)	ns	
No	97.6 (2490)	97.0 (592)	1		

Crude percentages and independent determinants for being a newly reached participant in univariate and multivariable analyses. Analyses were corrected for age, sex, and year of invitation. Restricted analyses were corrected for age, sexual preference, number of sex partners, and year of invitation.

### Proportion chlamydia positive tests

The proportion of chlamydia positive tests was comparable in newly reached participants (4.8%, 204/4298) versus participants previously tested at an STI clinic (3.6%, 11/304), by GPs (5.2%, 17/328), by medical specialists (3.7%, 7/187), or by a combination of providers (5.3%, 11/206) (P = 0.82). Overall, the proportion chlamydia positive tests was highest for the young participants (<21 years, 5.7%; age 22 to 24 years, 5.4%; age 25-29 years, 3.3%; P = 0.001).

## Discussion

This study assessed the totality of chlamydia tests in one geographic region by matching all chlamydia testing data to the municipal population register, including testing by systematic population-based chlamydia screening, the STI clinic, GPs and medical specialists. The chlamydia screening programme predominantly addressed new, previously untested, young participants who were not reached by regular care (81%) and who had similar urogenital chlamydia prevalence as participants previously tested elsewhere (4.8% vs. 4.5%). Therefore, we can conclude that the chlamydia screening programme adds to the existing regular care by revealing a so-far hidden population.

In this study, all chlamydia tests were collected: by regular care (GPs, STI clinic and medical specialists) and by intervention (chlamydia screening). There was no specific promotion of chlamydia testing during the chlamydia screening period that could have biased our results [5]. Previous studies also matched data from sexually transmitted infections/medical conditions diagnosed in several care settings to assess the proportion positives [11], births [14], ectopic pregnancies [14],[15], and reproductive capacity [15] after a chlamydia test. Our study adds to these previous studies by using the municipal population register which covers the entire population young people in one geographic region. This enabled assessing the totality of chlamydia testing, which was previously unknown [10].

Previous studies analysed participants in the English National Chlamydia Screening Programme (NCSP) and part of the Dutch systematic chlamydia screening programme. The NCSP is an opportunistic screening programme that offers chlamydia screening to eligible, sexually active individuals younger than 25 years when they attend consultations at specified health care, or other, settings [16]. Socio-demographic factors associated with participation in England were female gender and younger age [17]. Moreover, coverage was higher in deprived populations [18]. In the urbanised regions in the Netherlands, excluding this South Limburg study area, these factors were female gender and older age [8]. Separate analyses of South Limburg data gave similar results: female gender and older age were associated with participation (data not shown). In this study, newly reached participants turned out to be more often men, young (<21 years), and had had fewer sex partners compared to participants previously tested by regular care. This is important since men are usually harder to reach in screening programmes [16]. A possible explanation for this could be that men prefer testing in non-clinical settings such as postal testing kits and internet based screening [19]. In England only 15% of young men were tested for chlamydia last year in contrast to 35% of young women [20]. A qualitative study examining the barriers and facilitators of offering chlamydia testing in general practitioners (GPs) and practice nurses revealed that women have more consultations and it is easier to raise sexual health issues within the type of consultations women are seeking. Moreover, awkwardness and embarrassment were reported in raising chlamydia screening with men [21]. Another study found that GPs are reluctant to test young people for chlamydia in absence of urogenital symptoms [22]. Altogether, internet based screening could be helpful to stimulate especially young men [23]. New e-health strategies could provide more insight in the sexual and testing behaviour of young people [24].

Younger age is known to be associated with testing positive for chlamydia [8],[20], this makes young people a target group to reach in chlamydia screening. New participants had fewer sex partners, an explanation could be that people with more sex partners are more likely to attend STI clinics [25].

The chlamydia prevalence of newly reached participants was comparable to that of participants previously tested by regular care, and groups tested in other healthcare settings such as the STD clinic [26] and GPs [27]. This indicates that the chlamydia screening programme complements regular care in detecting chlamydia positives.

Sex was associated with being a newly reached participant in overall analyses, but not in additional analyses on questionnaire data. A possible explanation could be that a smaller proportion of men filled in the questionnaire compared to women. Being MSM was associated with being a newly reached participant, although this key population needs additional extra genital testing to fully address their risk behaviour [26]. Chlamydia testing was limited to urogenital site, which might underestimate the true burden of chlamydia as other studies showed that prevalence of anorectal chlamydia is substantial in both MSM and women [26],[28],[29].

This study has several limitations. First, it consists of not 100% uniquely matched data but 91%. The last 9% was matched using test records with identical markers from GPs and medical specialists. This slight inaccuracy might have introduced some bias and could lead to an underestimation of the proportion new participants. However, we expect this bias to be negligible as a sensitivity analysis on uniquely matched data revealed similar results. Unfortunately, data from care providers were only available for a restricted timeframe (2006-2010), limiting our results. It is possible that newly reached participants could have visited regular care before 2006.

Another limitation is that additional data on sexual behaviour was only available for the group that filled in extra self-administered questionnaires, which could lead to bias for the restricted data analysis. However, odds ratios for age were similar for the main analysis and the restricted data analysis (see Table 1), indicating that bias was unlikely.

The study area itself was another limitation. The eastern South Limburg region in the Netherlands is the first study area with sufficient and matchable data sources that can be used to assess the totality of chlamydia testing. However, this region is partly rural and it is unknown where data can be extrapolated to other participation regions that are more urban, such as Amsterdam and Rotterdam. We have to acknowledge that the generalizability of our results might be confined as screening programmes in different countries vary widely because of differences in recruitment methods, sample frames and participation rates. Moreover, not all persons in eastern South Limburg requesting chlamydia testing through the website were eligible due to the individuals’ chlamydia risk score. This may have led to higher positivity rates compared to systematic screening [12]. In a postal non-response study, no indications were found that participation in the screening was hampered by limited access to the internet. Non response to the screening was largely based on perceptions of individual risk [6].

## Conclusions

This study contributes to understanding the impact of chlamydia screening in reaching previously untested young people. In this study, chlamydia screening reached a hidden key population of young men and women who had never been tested before and who as a group showed a prevalence of chlamydia comparable to clinical settings.

## Authors’ original submitted files for images

Below are the links to the authors’ original submitted files for images. Authors’ original file for figure 1
