# Supplementary figures and images for: The added value of chlamydia screening between 2008-2010 in reaching young people in addition to chlamydia testing in regular care; an observational study
Source: BMC Infect Dis. 2014 Nov 18;14:612. doi: 10.1186/s12879-014-0612-2 (PMC4239384; doi:10.1186/s12879-014-0612-2)

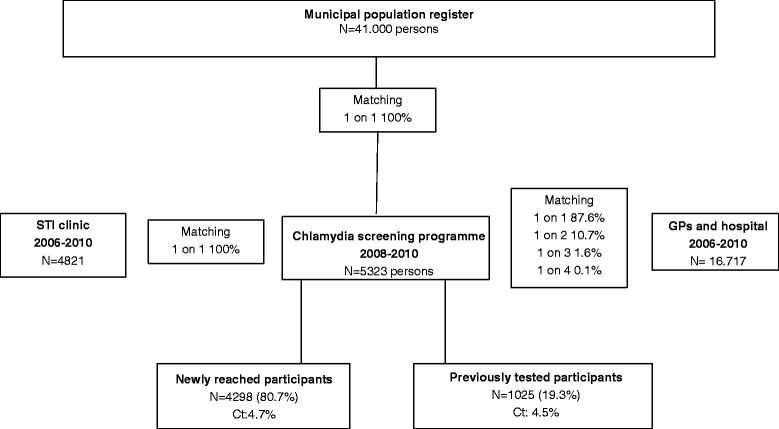

Supplement: Supplementary file 1 — Authors’ original file for figure 1 [file 12879_2014_612_MOESM1_ESM.gif]
